# A Hierarchical OhrR–EmrR Regulatory Circuit Fine‐Tunes the Vfm Quorum‐ Sensing System and Virulence in *Dickeya oryzae*

**DOI:** 10.1111/mpp.70354

**Published:** 2026-10-19

**Authors:** Wenqi Huang, Ziwei Meng, Zhongge Liu, Weihan Gu, Ming Hu, Tao Zhuo, Lianhui Zhang, Mingfa Lv

**Affiliations:** ^1^ College of Plant Protection Fujian Agriculture and Forestry University Fuzhou Fujian China; ^2^ Integrative Microbiology Research Centre South China Agricultural University Guangzhou Guangdong China

**Keywords:** *Dickeya oryzae*, OhrR‐EmrR, transcriptional regulator, Vfm quorum sensing, virulence

## Abstract

Rice foot rot, caused by *Dickeya oryzae*, is a destructive bacterial disease. The Vfm quorum‐sensing (QS) system is a key virulence regulator of this pathogen. However, the mechanisms by which it perceives and integrates upstream environmental signals for precise transcriptional control are poorly understood. Here, we identify a cascade regulatory network involving the MarR‐family transcription factors OhrR and EmrR. Deletion of *ohrR* in *D. oryzae* EC1 significantly reduced the Vfm signalling activity, diminished the production of virulence factors including phytotoxin zeamines and plant cell wall‐degrading enzymes, and attenuated its pathogenicity. Molecular analyses revealed that OhrR directly bound to the key promoters in the *vfm* cluster (P_*vfmA*_, P_*vfmK*_, P_*vfmE*_ and P_*vfmF*_) and activated their transcription. Intriguingly, OhrR also directly activated the expression of *emrR*, which encodes another MarR‐family transcriptional regulator. In contrast to the activator role of OhrR, EmrR acted as a feedback inhibitor, directly bound to the same *vfm* promoter regions but repressed their transcription. OhrR–EmrR thus acts as a two‐tiered ‘activation–inhibition’ regulatory circuit in modulation of the Vfm QS system, in which OhrR functions as a master switch for environmental signal response and initiation of the Vfm system, whereas EmrR acts as a precise brake to prevent overexpression of virulence determinants. The findings from this study unveil the fascinating interaction between OhrR–EmrR and the Vfm QS system, and depict a novel mechanism for precise virulence control in *D. oryzae* via complex, dynamic transcriptional reprogramming.

## Graphical abstract

We report a novel hierarchical regulatory circuit consisting of two MarR‐family transcription factors, OhrR and EmrR, that act antagonistically to dynamically fine‐tune the Vfm QS system and bacterial virulence.

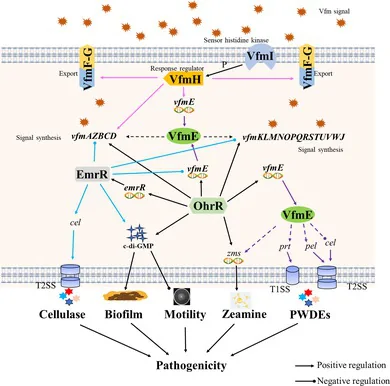

## 1 Introduction

Quorum sensing (QS), a process whereby bacterial cells communicate to coordinate gene expression and adapt to environmental changes, is now a well‐characterized phenomenon (Whitehead et al. 2001; Fuqua and Greenberg 2002; Federle and Bassler 2003). QS systems play pivotal roles in diverse bacterial species, regulating processes such as secondary metabolite biosynthesis and virulence‐related functions, including exopolysaccharide (EPS) production, antibiotic/toxin synthesis, biofilm formation, extracellular enzyme secretion and motility (Ohtani et al. 2002; Pemberton et al. 2005; Deng et al. 2010, 2011, 2012; Passos‐da‐Silva et al. 2017). A typical QS system consists of a signal synthase and a signal receptor, such as LuxI and LuxR in *Vibrio fischeri* and LasI and LasR in *Pseudomonas aeruginosa* (Fuqua and Greenberg 2002). At low bacterial cell density, LuxI or its homologues produces a basal level of QS signal acylhomoserine lactone (AHL) molecules that is not sufficient to activate the signal receptor LuxR or its homologues. When the bacterial population reaches a threshold level, the accumulated AHL signals interact and activate the receptor LuxR or its homologues, which consequently initiate the transcription of the downstream target genes. By using such a simple and yet elegant population density‐dependent QS mechanism, bacterial pathogens can mount their attack collectively to overwhelm host defence responses and establish infections (Whitehead et al. 2001; Fuqua and Greenberg 2002; Lee and Zhang 2015). However, evidence has emerged that bacterial pathogens can also subtly tap on host stress signals to fine‐tune QS output and modulate virulence gene expression (Lee et al. 2013).

*Dickeya oryzae* (formerly *Erwinia chrysanthemi* pv. *zeae*) is the causal agent of bacterial foot rot, causing significant economic losses in rice and banana production (Samson et al. 2005). It is one of the few *Dickeya* species with a broad host range, infecting both monocotyledonous and dicotyledonous plants (Sinha and Prasad 1977; Nassar et al. 1994). Similar to the well‐studied model species *Dickeya dadantii* (Diolez and Coleno 1985; Reverchon and Nasser 2013), genomic and biochemical studies of *D. oryzae* have identified conserved virulence factors, including plant cell wall‐degrading enzymes (PCWDEs), biofilm formation, EPS, an iron assimilation system, the antioxidant pigment indigoidine and a type III secretion system (T3SS) (Enard et al. 1988; Collmer and Bauer 1994; Reverchon et al. 2002; Yang et al. 2002; Yap et al. 2005; Zhou et al. 2015). Additionally, a family of phytotoxins/antibiotics termed zeamines constitutes a key virulence determinant that appears specific to *D. oryzae* and some *Dickeya solani* strains (Wu et al. 2010; Zhou et al. 2011; Cheng et al. 2013; Zhou et al. 2015, 2024). Furthermore, bacterial motility is also known to play a key role in *D. oryzae* systemic infection (Chen et al. 2020).

Recent research shows that virulence factor production in *D. oryzae* is controlled by several QS systems. Among them, the AHL‐based QS system influences motility and biofilm formation (Hussain et al. 2008). Putrescine, synthesized by SpeA of either bacterium or plant origin, also acts as a QS signal or a cross‐kingdom signal affecting motility, biofilm and virulence (Shi et al. 2019). The Vfm QS system, which is independent of the AHL‐based QS system, is crucial for the production of zeamine and PCWDEs, and essential for bacterial virulence (Lv et al. 2019; Zhou et al. 2024). Collectively, these findings underscore the important role of QS in *D. oryzae* EC1 virulence. Furthermore, the Vfm QS plays a dominant role in virulence regulation (Lv et al. 2019). However, the upstream regulatory mechanisms and components integrating environmental cues into these QS networks remain largely unknown.

The MarR‐family transcriptional regulator OhrR (organic hydroperoxide reductase regulator) is critical for several virulence associated traits in *D. oryzae* , including zeamine production, cellulase secretion, biofilm formation and motility (Lv et al. 2022). Another MarR‐family member, EmrR (multidrug efflux transporter repressor), similarly influences cellulase secretion, biofilm formation and motility (Lv et al. 2026). Both OhrR and EmrR belong to the MarR family, and their respective mutants show significant virulence attenuation. These observations suggest potential interconnections between OhrR, EmrR and the Vfm QS system, but the nature of these relationships is unclear. In this study, we demonstrated that in *D. oryzae* , OhrR directly and positively regulates the biosynthesis of Vfm signals, while EmrR directly and negatively regulates Vfm biosynthesis. We also showed that OhrR directly activates *emrR* transcription. These findings unveil a novel hierarchical regulatory circuit modulating QS and virulence in *D. oryzae* .

## 2 Results

### 2.1 OhrR Is Required for the Full Vfm QS Signalling Activity

Previous work showed that *ohrR* mutation results in reduced zeamine production and cellulase activity (Lv et al. 2022), and Vfm QS system mutants are impaired in zeamine and PCWDE production (Lv et al. 2019). To determine if OhrR affects Vfm QS signalling activity, we constructed the reporter strain ∆*vfmE*(*uidA*) to monitor the Vfm signal production. The reporter strain was co‐cultured separately with wild‐type EC1 or the strain ∆*ohrR* at a 1:1 ratio, and the bacterial growth (OD_600_) and fluorescence (β‐glucuronidase reporter [GUS] using 4‐methylumbelliferyl *β*‐D‐glucuronide as the substrate) were monitored simultaneously. The growth kinetics of strain EC1 and the strain ∆*ohrR* in co‐culture were comparable. Interestingly, the GUS fluorescence (normalized to cell density) was significantly lower in the co‐cultures containing the ∆*ohrR* compared to those with wild‐type EC1 (Figure 1), indicating that OhrR is required for the full Vfm QS signal production.

**FIGURE 1:**
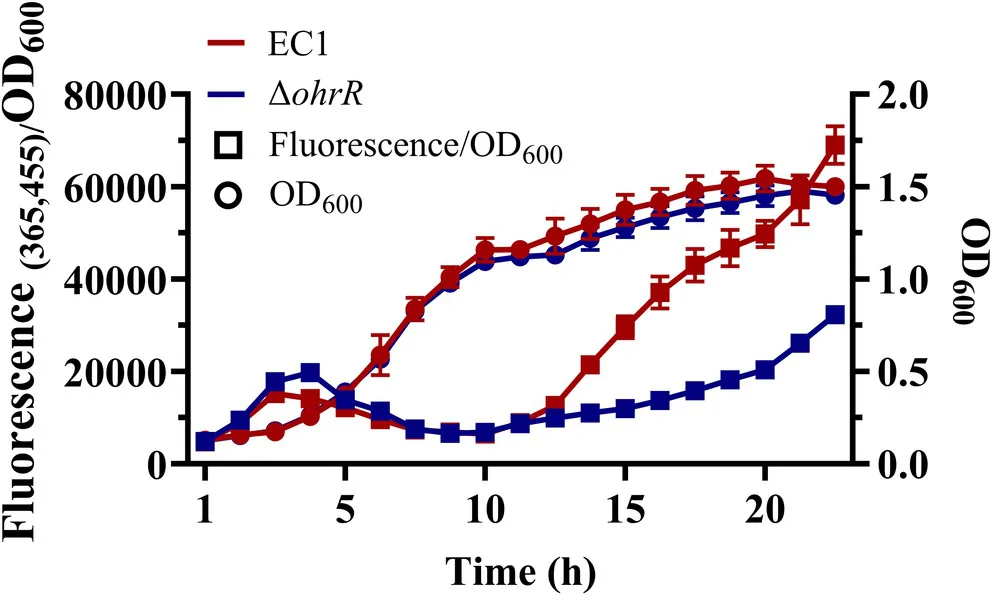
OhrR is required for the full Vfm quorum‐sensing (QS) signalling activity. Dynamics of Vfm QS signalling activity in co‐culture. The reporter strain ∆*vfmE*(*uidA*) was co‐cultured at a 1:1 ratio with wild‐type EC1 (red lines) or the ∆*ohrR* mutant (blue lines). Growth (OD_600_, circles) and normalized GUS fluorescence (fluorescence/OD_600_, squares) were monitored. Data are the representative of three independent experiments.

### 2.2 OhrR Positively Regulates Expression of the *vfm* Cluster

Because Vfm signal production was reduced in the strain ∆*ohrR*, we examined its effect on the expression of key *vfm* cluster operons. The promoter regions of the signal synthesis genes (*vfmA* and *vfmK*), the AraC‐family regulator gene (*vfmE*) and a signal transport gene (*vfmF*) (Figure 2a) were fused to the GFP gene in plasmid pROBE‐NT, generating transcriptional fusion reporter constructs P*vfmA‐gfp*, P*vfmK‐gfp*, P*vfmE‐gfp* and P*vfmF‐gfp*, and introduced into wild‐type EC1 and the ∆*ohrR*. GFP fluorescence measured at OD_600_ = 1.0 was significantly lower in the ∆*ohrR* background for all four promoters compared to the wild‐type background (Figure 2b). Consistent with this, reverse transcription‐quantitative PCR (RT‐qPCR) analysis confirmed that transcript levels of *vfmA*, *vfmE*, *vfmF* and *vfmK* were significantly reduced in the mutant ∆*ohrR* compared to wild‐type EC1 (Figure 2c). These results indicate that OhrR positively regulates the transcription of key operons within the *vfm* cluster.

**FIGURE 2:**
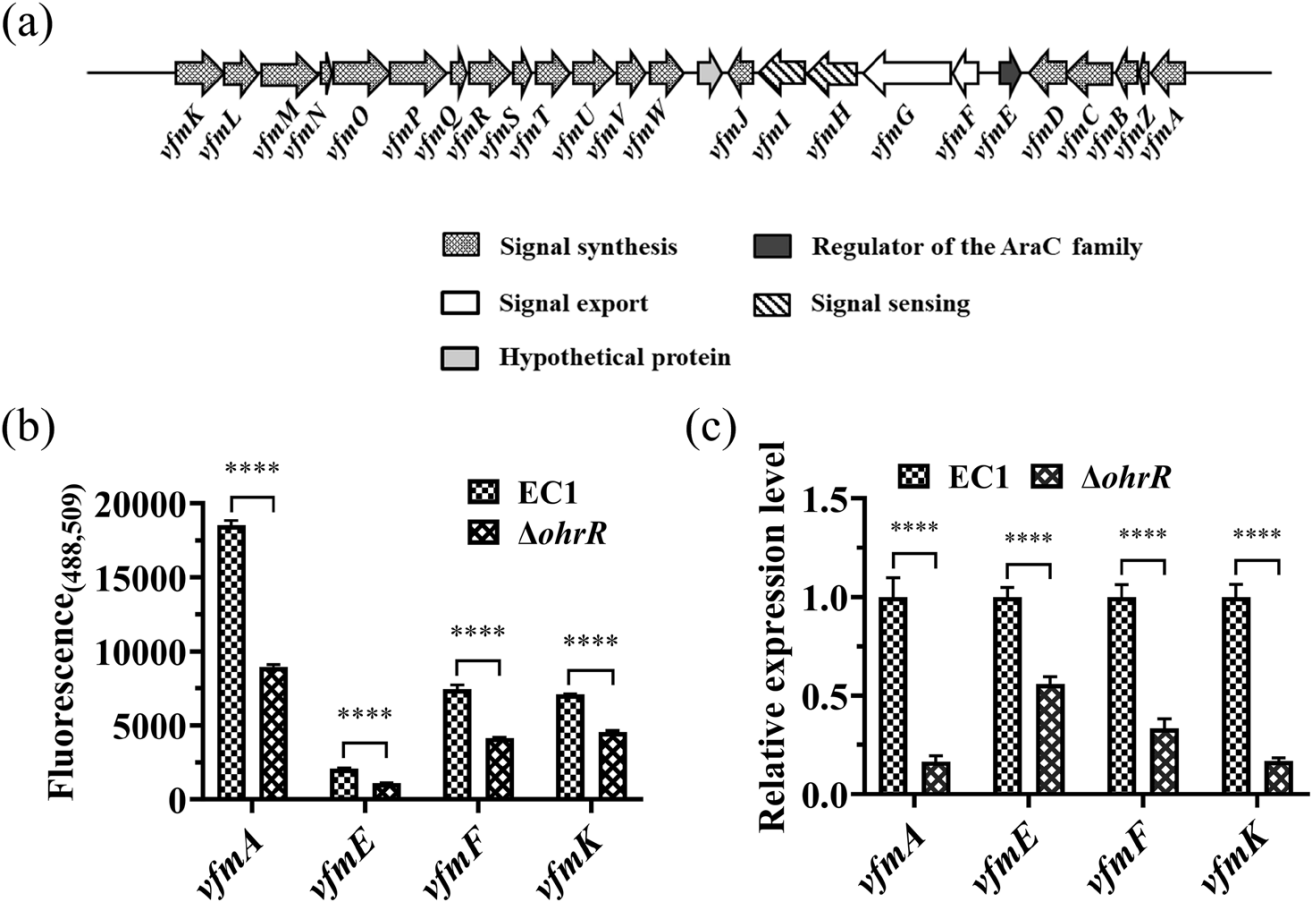
OhrR positively regulates expression of the *vfm* cluster. (a) Gene organization of the *vfm* gene cluster. (b) Promoter activity of key *vfm* operons. GFP fluorescence from transcriptional fusions (P*vfmA*‐*gfp*, P*vfmK*‐*gfp*, P*vfmE*‐*gfp* and P*vfmF*‐*gfp*) was measured in wild‐type EC1 and the ∆*ohrR* mutant at OD_600_ = 1.0. (c) Reverse transcription‐quantitative PCR analysis of *vfmA*, *vfmE*, *vfmF* and *vfmK* transcript levels in wild‐type EC1 and the ∆*ohrR* mutant. Expression was normalized to 16S rRNA and is shown relative to wild‐type EC1 (set to 1). Experiments were repeated at least three times in triplicate and error bars indicate stand deviation. ****p < 0.0001, Student's *t‐*test.

### 2.3 OhrR Directly Binds to the Promoters of *vfm* Genes

Given the reduced Vfm signal and gene expression in the strain ∆*ohrR*, we investigated whether OhrR directly regulates the *vfm* cluster. Recombinant OhrR protein was expressed in *Escherichia coli* and purified (Figure 3a). Electrophoretic mobility shift assays (EMSAs) showed that purified OhrR specifically bound to the promoter fragments of *vfmA*, *vfmE*, *vfmF* and *vfmK*, causing a mobility shift in a concentration‐dependent manner (Figure 3b and Figure S1). Bioinformatic analysis identified a conserved 20‐bp putative OhrR‐binding motif in these promoter regions (Figure 3c and Figure S2). EMSAs with synthesized oligonucleotides containing these motifs (B_*vfmA*_, B_*vfmE*_, B_*vfmF*_ and B_*vfmK*_) confirmed that OhrR directly and specifically bound to this conserved sequence (Figure 3d). These data demonstrate that OhrR directly activates the *vfm* gene expression by binding to a specific promoter motif.

**FIGURE 3:**
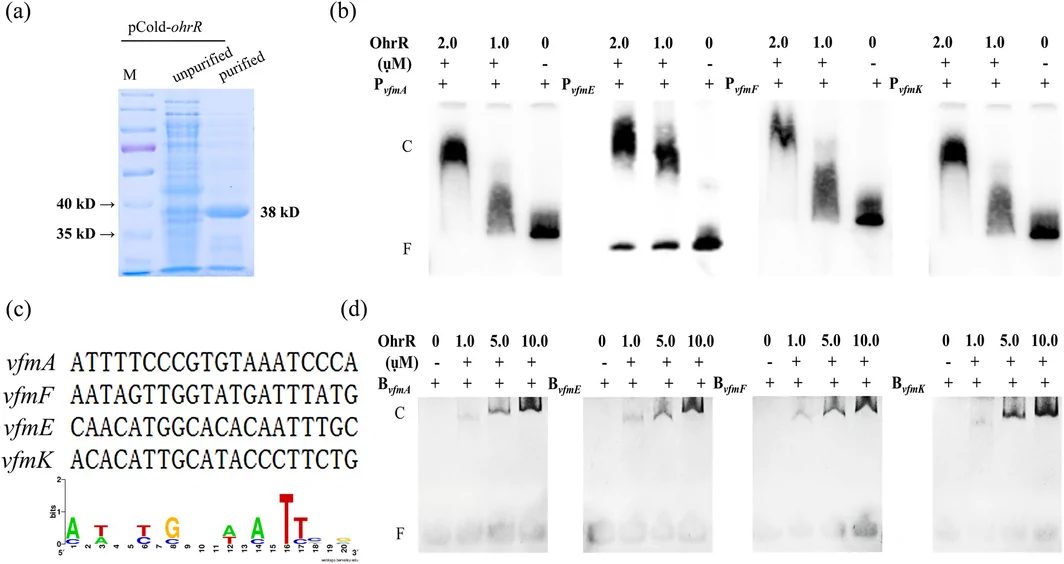
OhrR directly binds to the promoters of the *vfm* genes. (a) Purification of recombinant His‐tagged OhrR protein analysed by SDS‐PAGE. (b) Electrophoretic mobility shift assay (EMSA) showing binding of purified OhrR to the promoter fragments of *vfmA*, *vfmE*, *vfmF* and *vfmK*, with increasing concentrations of OhrR protein (0, 1, 2 μM) being used. (c) Sequence of the conserved OhrR‐binding motif identified in the promoters of *vfmA*, *vfmE*, *vfmF* and *vfmK*. (d) EMSA validation using synthesized oligonucleotides containing the predicted OhrR‐binding motifs (B_*vfmA*_, B_*vfmE*_, B_*vfmF*_ and B_*vfmK*_).

### 2.4 OhrR Directly Activates the Expression of *emrR*

As both OhrR and EmrR are MarR‐family regulators affecting virulence traits, we explored their potential regulatory relationship. The *emrR* mutant is defective in motility and biofilm formation, and EmrR autoregulates its own expression (Lv et al. 2026). We measured the promoter activity of *ohrR* and *emrR* using *lacZ* transcriptional fusions. The activity of the *ohrR* promoter was unaffected in a ∆*emrR* background (Figure 4a). In contrast, the activity of the *emrR* promoter was significantly lower in the strain ∆*ohrR* than wild‐type EC1 (Figure 4b). RT‐qPCR confirmed that *emrR* transcript level was reduced approximately 2.4‐fold in the mutant strain ∆*ohrR* (Figure 4c). Similarly, we also assessed *ohrR* promoter activity in the Δ*ohrR* and wild‐type strain EC1, and observed that *ohrR* promoter activity was markedly decreased in the Δ*ohrR* background (Figure S3). Bioinformatic analysis identified a conserved OhrR‐binding motif in the *emrR* promoter region (Figure 4d). EMSA confirmed that purified OhrR protein directly bound to this motif (Figure 4e). These results demonstrate that OhrR directly and positively regulates the transcription of *emrR*.

**FIGURE 4:**
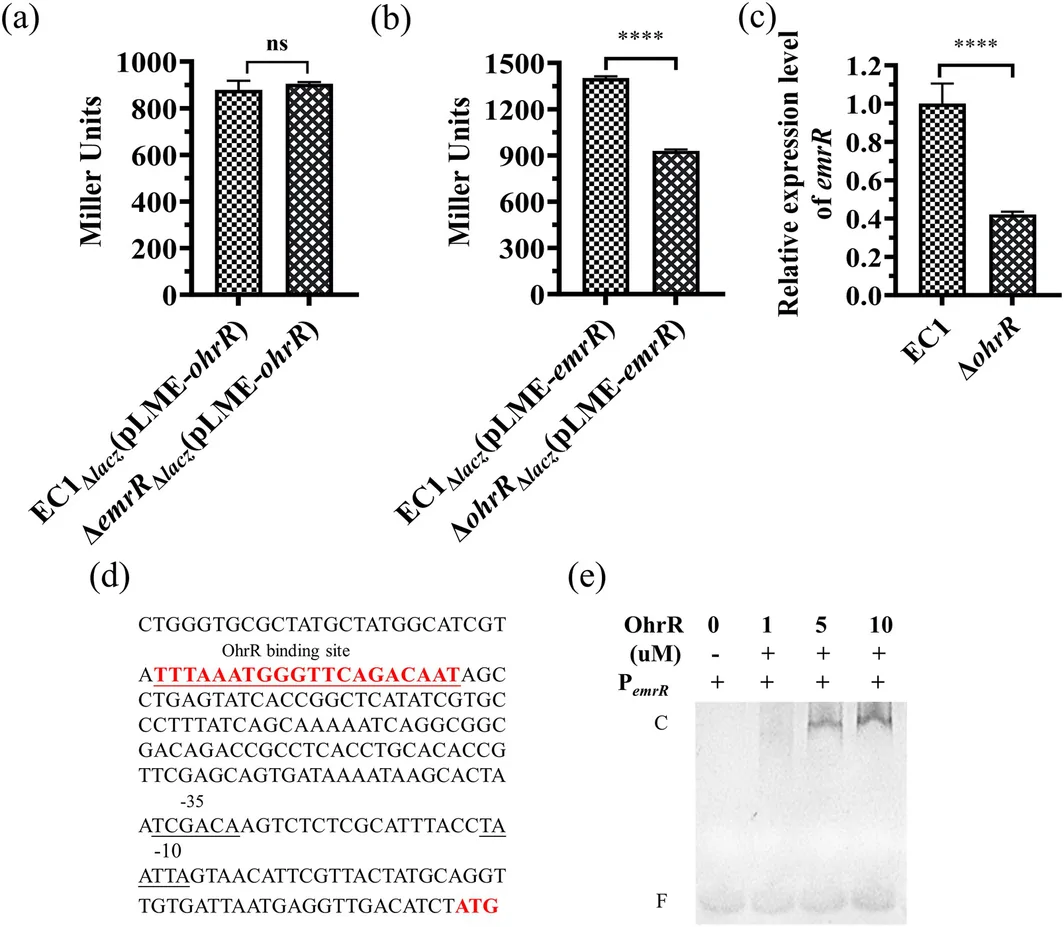
OhrR directly activates the transcription of *emrR*. (a, b) *β*‐Galactosidase activity assays. Promoter activity of (a) *ohrR* and (b) *emrR* was measured in the indicated genetic backgrounds using *lacZ* transcriptional fusions. (c) Reverse transcription‐quantitative PCR analysis of *emrR* expression in wild‐type EC1 and the ∆*ohrR* mutant. (d) Sequence of the predicted OhrR‐binding motif in the *emrR* promoter region. (e) Electrophoretic mobility shift assay confirming the direct binding of purified OhrR protein to the biotin‐labelled oligonucleotide containing the predicted *emrR* promoter motif. In (a–c), data are the means ± SD from three biological replicates. ****p < 0.0001, Student's *t‐*test.

### 2.5 EmrR Directly Represses the Vfm QS System

Because OhrR activates both the Vfm system and *emrR* expression, we next asked if EmrR could modulate the Vfm signal production. Using the ∆*vfmE*(*uidA*) reporter strain in a co‐culture assay, we found that Vfm signalling activity was significantly higher in the presence of the mutant strain ∆*emrR* compared to wild‐type EC1, despite similar growth curves (Figure 5a). Promoter‐GFP fusion assays showed that the activities of P_*vfmA*_, P_*vfmK*_, P_*vfmE*_ and P_*vfmF*_ were all elevated in the ∆*emrR* background compared to the wild type (Figure 5b). RT‐qPCR confirmed the increased transcript levels of these *vfm* genes in the mutant strain ∆*emrR* (Figure 5c). Recombinant EmrR protein was purified (Figure 5d). A conserved 21‐bp EmrR‐binding motif was identified in the promoters of *vfmA*, *vfmE*, *vfmF* and *vfmK* (Figure 5e), and EMSA verified that EmrR specifically and directly bound to this motif (Figure 5f and Figure S4). These findings establish EmrR as a direct negative regulator of the *vfm* gene cluster.

**FIGURE 5:**
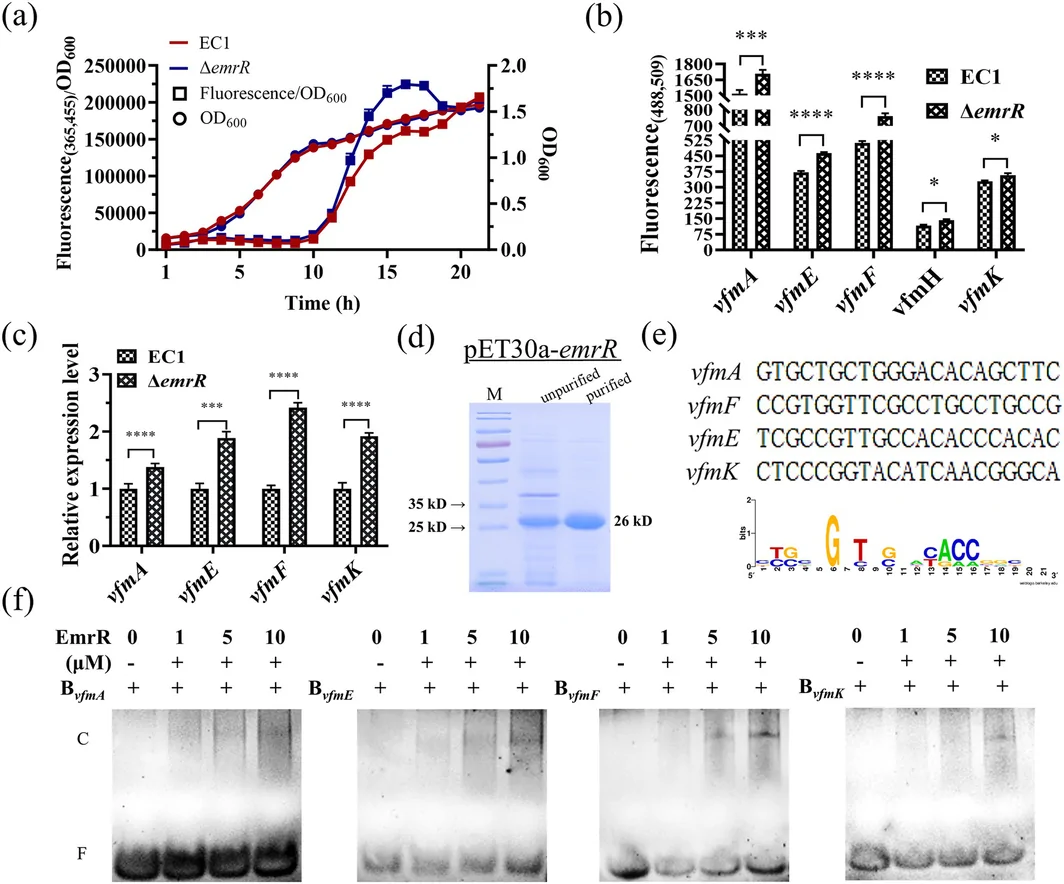
EmrR directly represses the Vfm quorum‐sensing (QS) system. (a) Vfm signal activity in co‐culture of the ∆*vfmE*(*uidA*) reporter with wild‐type EC1 (red lines) or the ∆*emrR* mutant (blue lines). (b) Promoter activity of the *vfm* genes (P*vfmA*‐*gfp*, P*vfmK*‐*gfp*, P*vfmE*‐*gfp* and P*vfmF*‐*gfp*) in wild‐type EC1 and the ∆*emrR* mutant. (c) Reverse transcription‐quantitative PCR (RT‐qPCR) analysis of *vfm* gene expression in the ∆*emrR* mutant relative to wild‐type EC1. (d) Purification of recombinant His‐tagged EmrR protein (SDS‐PAGE). (e) Candidate EmrR‐binding motif identified in the promoters of *vfmA*, *vfmF*, *vfmE* and *vfmK*.  (f) Electrophoretic mobility shift assay (EMSA) showing binding of purified EmrR to biotin‐labelled oligonucleotides containing the predicted binding motif. All experiments were conducted in triplicate and independently replicated at least three times. In (a–c), data are the means ± SD from three biological replicates. *p < 0.1, ***p < 0.0001, ****p < 0.0001, Student's *t‐*test.

### 2.6 Phenotypic Analysis of the *ohrR* and *emrR* Double Mutant

Given the opposing effects of OhrR (activator) and EmrR (repressor) on the Vfm system, we constructed a double‐deletion strain ∆*ohrRemrR* to assess their combined impact. The double mutant ∆*ohrRemrR* showed a more severe reduction in extracellular cellulase activity than either single‐mutant strain (∆*ohrR* and ∆*emrR*) (Figure 6a). Biofilm formation was significantly impaired in all three mutants (∆*ohrR*, ∆*emrR* and ∆*ohrRemrR*) to a similar degree (Figure 6b). Swimming motility was increased in ∆*ohrR*, decreased in ∆*emrR*, and restored to near wild‐type levels in the double‐mutant strain ∆*ohrRemrR* (Figure 6c). The single‐mutant strain ∆*emrR* maintained wild‐type strain EC1 zeamine levels. Zeamine production was significantly decreased in the mutant strain ∆*ohrR*, and the double‐deletion mutant strain ∆*ohrRemrR* displayed zeamine levels comparable to the single‐mutant strain ∆*ohrR* (Figure 6d). The potato slice inoculation test demonstrated that, in comparison with the single‐mutant strain ∆*ohrR* and ∆*emrR*, the double‐deletion mutant strain ∆*ohrRemrR* exhibited a further diminished capacity to cause maceration on potato slices (Figure 6e,f). These complex phenotypic variations suggest that OhrR and EmrR form a regulatory circuit that finely tunes different virulence factors outputs, with EmrR providing feedback inhibition on the OhrR–Vfm axis.

**FIGURE 6:**
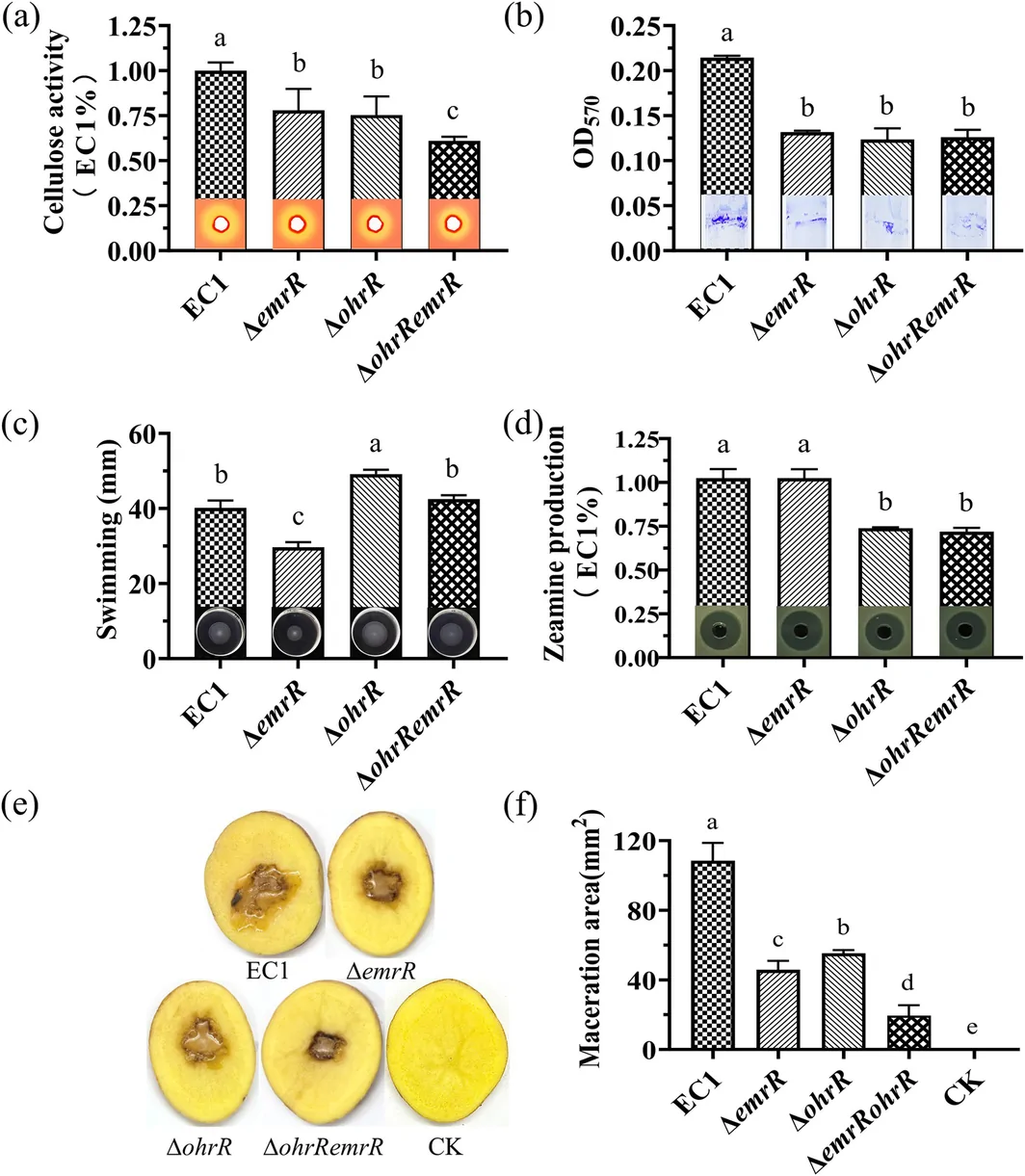
Phenotypic characterization of single and double *ohrR emrR* mutants. (a) Extracellular cellulase activity. (b) Biofilm formation (crystal violet staining). (c) Swimming motility. (d) Zeamine production. (e) Maceration of potato tuber slices by wild‐type EC1, ∆*ohrR*, ∆*emrR* and ∆*ohrRemrR* strains. Data are the means ± SD from three independent experiments. Different letters above bars indicate statistically significant differences (*p* < 0.05, one‐way ANOVA with post hoc multiple comparisons. Pairwise comparisons included wild‐type EC1 versus each mutant, and comparisons among Δ*ohrR*, Δ*emrR* and Δ*ohrRemrR* mutants. Different letters represent significant differences).

## 3 Discussion

This study reveals a precise transcriptional cascade in which the MarR‐family regulators OhrR and EmrR hierarchically and antagonistically modulate the Vfm QS system to control virulence in *D. oryzae* EC1. We demonstrate that OhrR directly activates the *vfm* gene cluster (Figures 1, 2, 3) and simultaneously induces the expression of *emrR* (Figure 4). EmrR then acts as a direct negative feedback regulator of the same *vfm* cluster (Figure 6). For the regulation of cellulase activity, OhrR and EmrR act congruently as negative regulators, with the double mutant exhibiting an additive effect (Figures 6 and 7). This ‘activation–inhibition’ dual‐layer regulatory circuit (Figure 7) provides a framework for understanding how plant pathogens achieve precise spatiotemporal control of virulence through complex transcriptional reprogramming.

**FIGURE 7:**
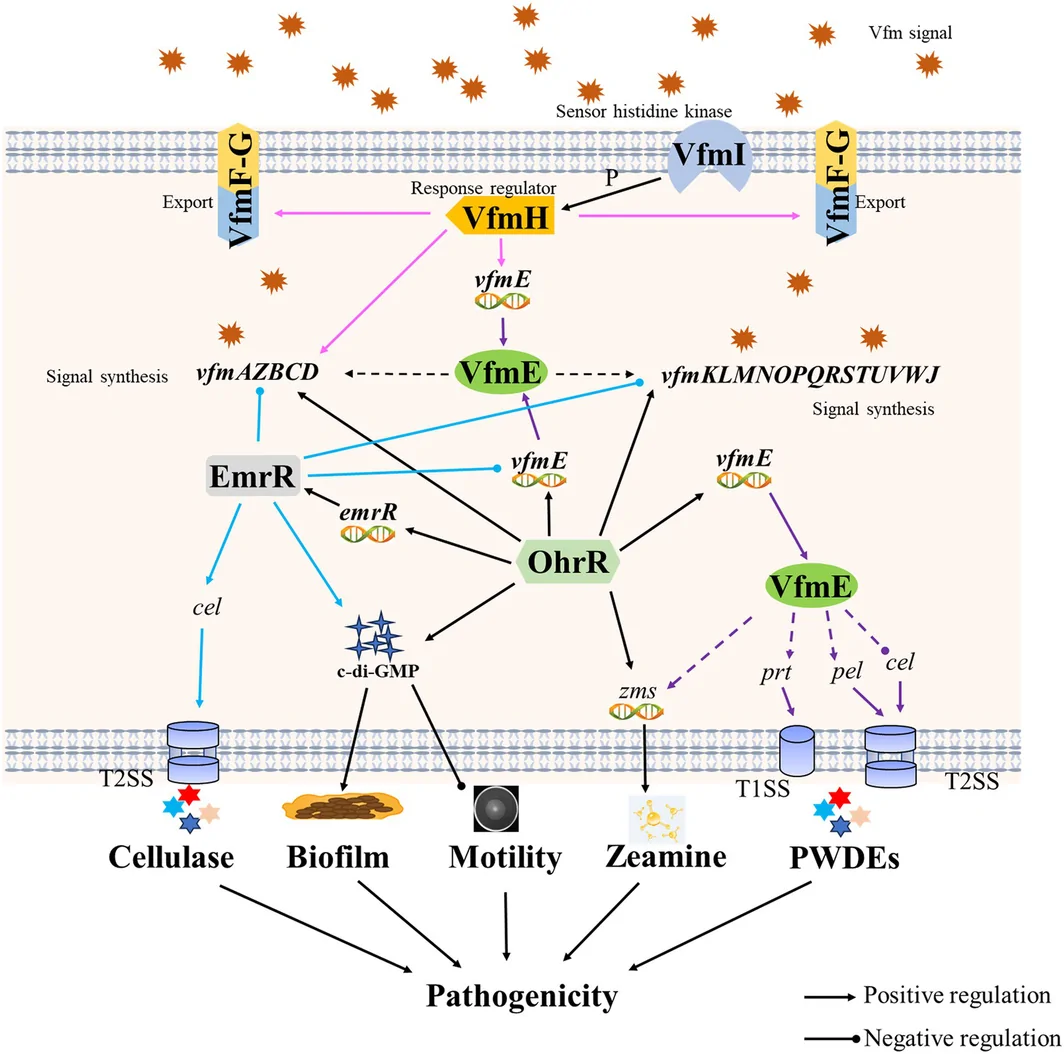
Schematic model of the OhrR–EmrR regulatory circuit modulating the Vfm quorum‐sensing (QS) system in *Dickeya oryzae*. OhrR, potentially activated by environmental signals (e.g., oxidative stress), directly binds to and activates the transcription of key operons within the *vfm* cluster (*vfmAZBCD*, *vfmKLMNOPQRSTUVW*, *vfmE* and *vfmFG*), promoting Vfm signal synthesis, regulation and transport. Simultaneously, OhrR directly activates the expression of *emrR*. EmrR protein then acts as a feedback inhibitor, directly binding to the same *vfm* promoter regions to repress their transcription, preventing over‐activation. This ‘activation–inhibition’ cascade allows for precise, dynamic control of the Vfm QS system and downstream virulence factor production. Dashed lines indicate the putative regulatory patterns that they have not yet been experimentally verified. PWDEs, plant cell wall‐degrading enzymes; T1SS, type I secretion system; T2SS, type II secretion system.

Notably, the predicted OhrR and EmrR binding motifs within the *vfm* promoters display relatively low sequence conservation across different target sites (Figures 3c and 5e). Poorly conserved or degenerate binding sequences are frequently observed among MarR family transcriptional regulators, and sequence conservation alone is insufficient to confirm authentic protein–DNA interactions (Nazaret et al. 2023). Our cross‐EMSA and competitive EMSA assays nevertheless support the specific binding of OhrR and EmrR to these *vfm* promoter fragments (Figures [Link], [Link] and S5), reinforcing that biochemical validation is critical to verify predicted binding sites when motif conservation is limited. Similar observations have been reported for other MarR type regulators, where functional binding sites deviate substantially from consensus motifs obtained from different bacterial species (Deochand and Grove 2017; Nazaret et al. 2023).

A central finding places OhrR and EmrR upstream of the previously characterized VfmIH–VfmE signal transduction axis (Nasser et al. 2013; Lv et al. 2019). OhrR acts as a master switch by directly binding to the promoters of genes for signal synthesis (*vfmA* and *vfmK*), regulation (*vfmE*) and transport (*vfmF*) to initiate and amplify the Vfm‐QS activity (Figure 3). The absence of OhrR results in a substantial collapse of Vfm signalling and bacterial virulence (Lv et al. 2022). A key feature of this model is the cascading regulatory relationship between OhrR and EmrR (Figure 7). Direct activation of *emrR* by OhrR establishes an intrinsic feedback loop. Accumulated EmrR subsequently dampens OhrR‐dependent activation by repressing the same set of *vfm* promoters. This regulatory architecture has two major functional consequences. First, it supports dynamic balance and pulsed virulence responses. Instead of a simple on–off switch, the circuit enables dynamically balanced, potentially pulsed virulence outputs. OhrR can respond to environmental oxidative cues (Duarte and Latour 2010; Garnica et al. 2017; Meireles et al. 2022) and rapidly trigger virulence gene expression. Concurrent induction of EmrR restrains metabolically costly virulence factor production, which may facilitate immune evasion or optimize resource allocation during later infection stages. Second, this module constitutes a signal integration mode. As typical MarR‐family sensors, OhrR likely perceives host‐derived stress signals such as reactive oxygen species (ROS) and integrates these inputs into the QS regulatory network. The OhrR–EmrR hierarchical cascade (Figure 7) therefore allows *D. oryzae* to fine‐tune virulence according to both population density and prevailing environmental conditions.

Our results, together with previous studies, highlight the complexity of QS regulation within *Dickeya*. First, there is considerable specificity and strain‐level variation. The Vfm signal molecules differ between *D. oryzae* and *D. dadantii* (Lv et al. 2019), and polymorphisms in *vfmO/P* generate strain‐specific signal analogues (Hugouvieux‐Cotte‐Pattat et al. 2022). Differences in *ohrR*/*emrR* expression, protein activity or binding site sequences among strains may further shape these strain‐specific regulatory patterns. Second, cross‐system interactions vary across species. In *D. solani*, the Vfm and AHL‐based Exp systems do not synergistically control PCWDEs or pathogenicity, and Vfm exerts a more dominant role than in other *Dickeya* species (Potrykus et al. 2018). Our findings on the internal fine‐tuning of the Vfm system emphasize its central importance, with dedicated regulatory modules such as OhrR–EmrR enabling precise output control. Third, Vfm is embedded within larger global regulatory networks. The Vfm system receives inputs from multiple global regulators including Fis, IHF and PecS (Nasser et al. 2013; Lv et al. 2018; Chen, Hu, et al. 2022; Chen, Li, et al. 2022). The addition of the redox sensing OhrR and its downstream EmrR expands our understanding of how *Dickeya* integrates diverse environmental signals to optimize virulence.

Beyond elucidating this novel regulatory mechanism, the OhrR–EmrR–Vfm cascade raises questions regarding its evolutionary advantage and its potential as a target for disease control strategies. The ‘activation–inhibition’ circuit likely provides fitness benefits within the dynamic host apoplast, where pathogens encounter fluctuating stressors such as ROS bursts associated with pathogen‐associated molecular pattern (PAMP)‐triggered immunity (Torres et al. 2006; Mittler et al. 2011). The OhrR–EmrR regulatory circuit may equip *D. oryzae* with a ‘measured aggression’ infection strategy. OhrR, which can sense oxidative stress (Fuangthong and Helmann 2002), triggers rapid virulence gene activation to establish infection and counteract host defences. OhrR‐dependent induction of EmrR then acts as an intrinsic dosage controller, preventing energetically wasteful overproduction of PCWDEs and toxins. Unrestricted production of these factors could trigger severe host tissue damage and activate systemic host defences (Boller and He 2009). Such fine‐tuning may facilitate transition from initial invasion to sustained colonization by balancing virulence investment with immune evasion. This layered regulatory logic is reminiscent of virulence circuits in animal bacterial pathogens, pointing toward convergent evolution of transcription‐based resilience for host adaptation (Galán 2021).

In summary, we have identified and characterized a cascade of transcriptional hierarchy involving OhrR and EmrR that might precisely and dynamically govern the central Vfm QS pathway in *D. oryzae* . OhrR serves as an environmental cue detector and system initiator, while EmrR functions as an internal feedback modulator. This discovery not only expands the functional repertoire of MarR regulators into the realm of bacterial cell–cell communication but, more importantly, reveals that plant pathogens employ multilayered, logic‐gated regulatory circuits far beyond simple density sensing to achieve optimal virulence expression.

## 4 Experimental Procedures

### 4.1 Bacterial Strains and Growth Conditions

Bacterial strains and plasmids used in this study are listed in Table 1. *E. coli* strains were cultured at 37°C, and *D. oryzae* EC1 and its derivatives were cultured at 30°C. Lysogeny broth (LB), minimal medium (MM) or LS5 medium (Cheng et al. 2013; Liao et al. 2014) were used as indicated. Antibiotics were used at the following concentrations: polymyxin B sulphate (PB, 25 μg/mL), streptomycin (Str, 50 μg/mL), kanamycin (Kan, 50 μg/mL) and ampicillin (Amp, 100 μg/mL).

**TABLE 1:** Strains and plasmids used in this study.

Strains or plasmids	Relevant phenotypes and characteristics^a^	Source or references
Strains		
EC1	Wild type of *Dickeya oryzae*, PB^r^	Hussain et al. (2008)
EC1_Δ*lacZ*_	*lacZ* in‐frame deletion mutant derived from EC1, PB^r^	This research
Δ*emrR*	*emrR* in‐frame deletion mutant derived from EC1, Pb^r^	Lv et al. (2026)
Δ*emrR*_Δ*lacZ*_	*emrR* in‐frame deletion mutant derived from EC1_Δ*lacZ*_, PB^r^	Lv et al. (2026)
Δ*ohrR*	*ohrR* in‐frame deletion mutant derived from EC1, PB^r^	Lv et al. (2022)
Δ*ohrR*_Δ*lacZ*_	*ohrR* in‐frame deletion mutant derived from EC1_Δ*lacZ*_, PB^r^	This research
Δ*ohrR*Δ*emrR*	*ohrR* and *emrR* double in‐frame deletion mutant from EC1, PB^r^	This research
EC1_Δ*lacZ*_ (pLME‐*emrR*)	The EC1_Δ*lacZ*_ was transformed with plasmid pLAME‐*emrR*, PB^r^, Amp^r^	This research
Δ*emrR*_Δ*lacZ*_ (pLME‐*ohrR*)	The Δ*emrR*_Δ*lacZ*_ was transformed with plasmid pLAME‐*ohrR*, PB^r^, Amp^r^	This research
EC1_Δ*lacZ*_ (pLME‐*ohrR*)	The EC1_Δ*lacZ*_ was transformed with plasmid pLAME‐*ohrR*, PB^r^, Amp^r^	This research
Δ*ohrR*_Δ*lacZ*_ (pLME‐*emrR*)	The Δ*ohrR*_Δ*lacZ*_ was transformed with plasmid pLAME‐*emrR*, PB^r^, Amp^r^	This research
EC1 (pROBE‐*vfmA*)	The EC1 was transformed with plasmid pROBE‐*vfmA*, PB^r^, Kan^r^	This research
EC1 (pROBE‐*vfmE*)	The EC1 was transformed with plasmid pROBE‐*vfmE*, PB^r^, Kan^r^	This research
EC1 (pROBE‐*vfmF*)	The EC1 was transformed with plasmid pROBE‐*vfmF*, PB^r^, Kan^r^	This research
EC1 (pROBE‐*vfmK*)	The EC1 was transformed with plasmid pROBE‐*vfmK*, PB^r^, Kan^r^	This research
Δ*ohrR* (pROBE‐*vfmA*)	The Δ*ohrR* was transformed with plasmid pROBE‐*vfmA*, PB^r^, Kan^r^	This research
Δ*ohrR* (pROBE‐*vfmE*)	The Δ*ohrR* was transformed with plasmid pROBE‐*vfmE*, PB^r^, Kan^r^	This research
Δ*ohrR* (pROBE‐*vfmF*)	The Δ*ohrR* was transformed with plasmid pROBE‐*vfmF*, PB^r^, Kan^r^	This research
Δ*ohrR* (pROBE‐*vfmK*)	The Δ*ohrR* was transformed with plasmid pROBE‐*vfmK*, PB^r^, Kan^r^	This research
Δ*emrR* (pROBE‐*vfmA*)	The Δ*emrR* was transformed with plasmid pROBE‐*vfmA*, PB^r^, Kan^r^	This research
Δ*emrR* (pROBE‐*vfmE*)	The Δ*emrR* was transformed with plasmid pROBE‐*vfmE*, PB^r^, Kan^r^	This research
Δ*emrR* (pROBE‐*vfmF*)	The Δ*emrR* was transformed with plasmid pROBE‐*vfmF*, PB^r^, Kan^r^	This research
Δ*emrR* (pROBE‐*vfmK*)	The Δ*emrR* was transformed with plasmid pROBE‐*vfmK*, PB^r^, Kan^r^	This research
EC1 (pROBE‐*ohrR*)	The EC1 was transformed with plasmid pROBE‐*ohrR*, PB^r^, Kan^r^	This research
Δ*ohrR* (pROBE‐*ohrR*)	The Δ*ohrR* was transformed with plasmid pROBE‐*ohrR*, PB^r^, Kan^r^	This research
Δ*vfmE* (*uidA*)	The reporter strain of VFM signal, PB^r^	This research
DH5α	*Escherichia coli* strain as host for plasmid constructs derived from pBBR1‐MCS4	Zhou et al. (2011)
HB101 (pRK2013)	*E. coli, thr leu thi recA hsdR hsdM pro*, Kan^r^	Zhou et al. (2011)
CC118λ	*E. coli* strain as host for plasmid constructs derived from pKNG101	Zhou et al. (2011)
BL21 (DE)	*E. coli* strain as host for protein prokaryotic expression plasmids	Zhou et al. (2011)
Plasmids		
pKNG101	Knockout vector, Str^r^	Zhou et al. (2011)
pLME	A promote probe vector that contains *lacZ*, Amp^r^	Lv et al. (2026)
pLME‐*emrR*	The promoter probe vector pLME was fused the promoter region of *emrR*, Amp^r^	This research
pLME‐*ohrR*	The promoter probe vector pLME was fused the promoter region of *ohrR*, Amp^r^	This research
pROBE‐NT	A promoter probe vector that contains *gfp*, Kan^r^	This research
pROBE‐*vfmA*	The promoter probe vector pROBE‐NT was fused the promoter region of *vfmA*, Kan^r^	This research
pROBE‐*vfmE*	The promoter probe vector pROBE‐NT was fused the promoter region of *vfmE*, Kan^r^	This research
pROBE‐*vfmF*	The promoter probe vector pROBE‐NT was fused the promoter region of *vfmF*, Kan^r^	This research
pROBE‐*vfmK*	The promoter probe vector pROBE‐NT was fused the promoter region of *vfmK* Kan^r^	This research
pROBE‐*ohrR*	The promoter probe vector pROBE‐NT was fused the promoter region of *ohrR* Kan^r^	This research
pCold‐SUMO‐M	Protein prokaryotic expression vector, Amp^r^	Liu et al. (2017)
pCold‐*ohrR*	pCold‐SUMO‐M carries the *ohrR* coding region, Amp^r^	This research
pET30a	Protein prokaryotic expression vector, Kan^r^	Lv et al. (2026)
pET30a‐*emrR*	pET30a carries the *emrR* coding region, Kan^r^	This research

^a^ PB^r^, Amp^r^, Kan^r^, Str^r^ = resistance to polymyxin B sulphate, ampicillin, kanamycin, and streptomycin, respectively.

### 4.2 Construction of Deletion Mutants

In‐frame deletion mutants were constructed using the suicide vector pKNG101 via triparental mating and *sacB*‐based counterselection as previously described (Lv et al. 2018, 2019). Flanking regions (approximately 600 bp) of the target genes were amplified with specific primers (Table S1) and sequentially cloned into pKNG101.

### 4.3 Construction and Measurement of GFP Reporter Strains

Promoter regions (approximately 300–500 bp upstream of the start codon) were amplified and cloned into the BamHI/HindIII sites of the GFP reporter vector pROBE‐NT. Recombinant plasmids were transferred into *D. oryzae* strains via triparental mating. Overnight cultures were diluted 1:100 in fresh LB and grown with shaking at 28°C. GFP fluorescence (excitation 488 nm, emission 509 nm) and OD_600_ were measured periodically using a microplate reader (BioTek). Data from at least three independent biological replicates are presented.

### 4.4 GUS Activity Assay for Vfm Signal Detection

A Vfm signal reporter strain, ∆*vfmE* (*uidA*), was constructed by inserting a promoterless *uidA* (GUS) gene into the *vfmE* locus (Lv et al. 2019). To measure signal activity *in trans*, culture supernatants (20% v/v final) from test strains were added to the reporter culture. For co‐culture assays, the reporter strain was mixed 1:1 with the test strain. Cultures containing 100 μg/mL 4‐methylumbelliferyl *β*‐D‐glucuronide (MUG) were incubated overnight. Fluorescence (excitation 365 nm, emission 455 nm) and OD_600_ were measured at the time points as indicated.

### 4.5 Detection of Extracellular Enzyme Activity

Qualitative assays of extracellular cellulase (Cel) activity were conducted on LB agar plates supplemented with 0.5% carboxymethyl cellulose sodium, as previously described (Chatterjee et al. 1995; Lv et al. 2018). Plates were incubated at 28°C for 16 h prior to staining with Congo red. Excess dye was washed off using 1 M NaCl solution, and the radius of each cellulolytic degradation halo was recorded.

### 4.6 Bioassay for Zeamine Production

Zeamine production was assessed using an *E. coli* DH5α growth inhibition assay on LS5 agar plates, following established protocols (Lv et al. 2018, 2019).

### 4.7 Motility and Biofilm Assays

Swimming motility was tested on 0.2% agar plates containing 1% tryptone and 0.5% NaCl. Plates were point‐inoculated and incubated at 28°C for 15 h before measuring migration diameter. Biofilm formation was quantified in static SOBG medium using the crystal violet staining method as described (Chen et al. 2020). All assays were performed with at least three biological replicates.

### 4.8 *β*‐Galactosidase Activity Assay

The promoter regions of *ohrR* and *emrR* were fused separately to *lacZ* in plasmid pLME (Lv et al. 2026). The resulting plasmids were introduced into the relevant *D. oryzae* ∆*lacZ* strain. *β*‐Galactosidase activity was measured in the cells grown to mid‐exponential phase (OD_600_ = 1.0) using *o*‐nitrophenyl‐*β*‐D‐galactopyranoside (ONPG) as a substrate as described (Meng et al. 2020; Lv et al. 2026). Activity is expressed in Miller units.

### 4.9 RNA Extraction and RT‐qPCR

Total RNA was extracted from bacterial cultures at OD_600_ = 1.0 using the SV Total RNA Isolation System (Promega). cDNA was synthesized using the FastQuant RT Kit (Tiangen). qPCR was performed with SuperReal PreMix Colour SYBR Green (Tiangen) on a QuantStudio 6 Flex system. The 16S rRNA gene was used as an internal reference. Gene expression changes were calculated using the 2^−∆∆*C*t^ method (Livak and Schmittgen 2001). Data represent at least two independent runs with four biological replicates each.

### 4.10 Protein Purification and EMSA

The coding sequences of *ohrR* and *emrR* were cloned into pCold‐SUMO‐M or pET‐30a vectors (Liu et al. 2017; Xu et al. 2023; Lv et al. 2026), respectively. Recombinant His‐tagged proteins were expressed in *E. coli* BL21 (DE3) and purified using Ni‐NTA resin (Clontech). Promoter DNA fragments or biotin‐labelled oligonucleotides were incubated with purified proteins in binding buffer for 30 min at room temperature. Protein–DNA complexes were resolved on 6% non‐denaturing polyacrylamide gels in 0.5× TBE buffer and detected using the Chemiluminescent Nucleic Acid Detection Module (Thermo Fisher Scientific).

### 4.11 Prediction of OhrR and EmrR Binding Motifs

Putative OhrR and EmrR binding sites in the promoter regions of *vfmA*, *vfmE*, *vfmF* and *vfmK* were predicted by sequence alignment using known motifs (implemented via Clustal Omega v. 6.0). The OhrR motif was the published *Bacillus subtilis* OhrR binding site (5′‐TTAATTGTGTACAATTTA‐3′; Fuangthong et al. 2001), and the EmrR seed was the *E. coli* EmrR consensus (5′‐CTGTCGTTACTATATCGGCTG‐3′; Xiong et al. 2000). Approximately 500 bp sequences upstream of each gene start codon were aligned against the seed sequences to retrieve candidate binding fragments. The obtained candidate sites were subsequently verified via EMSA. Sequence logos of our identified OhrR and EmrR motifs were generated using WebLogo v. 3.

### 4.12 Statistical Analysis

All experiments were performed with at least three independent biological replicates. Data are presented as mean ± SD. Statistical significance was determined using Student's *t*‐test or one‐way ANOVA with appropriate post hoc tests, as indicated in the figure legends. Differences were considered significant at *p* < 0.05. All analyses were performed using GraphPad Prism v. 8.0 software.

## Author Contributions

**Wenqi Huang:** investigation. **Ziwei Meng:** investigation. **Zhongge Liu:** investigation. **Weihan Gu:** methodology. **Ming Hu:** methodology. **Tao Zhuo:** formal analysis, methodology. **Lianhui Zhang:** writing – review and editing, methodology, funding acquisition. **Mingfa Lv:** writing – review and editing, writing – original draft, supervision, funding acquisition, formal analysis, project administration.

## Funding

This work was supported by grants from the National Natural Science Foundation of China (No. 32570212, U22A20480) and the Science Foundation of Fujian Provincial Science & Technology Department (2023J01440, 2022J01128).

## Conflicts of Interest

The authors declare no conflicts of interest.

## Supporting information


**Figure S1:** Specific competitive electrophoretic mobility shift assays (EMSAs) for OhrR and EmrR binding to the *vfmK* promoter, with the *pelE* promoter fragment serving as a negative control.

**Figure S2:** Localization of OhrR and EmrR binding motifs within the *vfmA*, *vfmE*, *vfmF* and *vfmK* promoter regions. Schematic representation showing the positions of identified OhrR and EmrR binding motifs upstream of the translation start sites of *vfmA*, *vfmE*, *vfmF* and *vfmK*.

**Figure S3:** *ohrR* promoter activity assay in wild‐type EC1 and Δ*ohrR*. *p* < 0.0001 (*t*‐test).

**Figure S4:** Cross‐electrophoretic mobility shift assay (EMSA) validation of binding specificity for OhrR and EmrR toward their respective sites in the *vfmA* promoter.

**Figure S5:** Electrophoretic mobility shift assay (EMSAs) using the *vfmA* promoter probe with OhrR and EmrR at different molar ratios.

**Table S1:** Primers used in this study.

## Data Availability

The data that support the findings of this study are available on request from the corresponding author. The data are not publicly available due to privacy or ethical restrictions.
