# Supplementary material for: A Hierarchical OhrR–EmrR Regulatory Circuit Fine‐Tunes the Vfm Quorum‐ Sensing System and Virulence in Dickeya oryzae
Source: Mol Plant Pathol. 2026 Oct 19;27(10):e70354. doi: 10.1111/mpp.70354 (PMC13680859; doi:10.1111/mpp.70354)
Supplement: Supplementary file 1 [file MPP-27-e70354-s001.docx]

**Table S1.** Primers used in this study

| Primers | Sequence (5'-3') | |
| --- | --- | --- |
| Mutant construction primers | |  |
| pROBE-*vfmA*-F | ggaattggggatcgg**aagctt**CGTTCATTGAGGACCACGGT | |
| pROBE-*vfmA*-R | gagctcggtacccgg**ggatcc**GGCATTCAGGCAAAACATGTC | |
| pROBE-*vfmE*-F | ggaattggggatcgg**aagctt**CCGCAAAACTGACTTCACCTTT | |
| pROBE-*vfmE*-R | gagctcggtacccgg**ggatcc**GGTTATTGTTAAAAATATCGTTATCTACGG | |
| pROBE-*vfmH*-F | ggaattggggatcgg**aagctt**CCTAAGCATTTGCTTAAAATGCTG | |
| pROBE-*vfmH*-R | gagctcggtacccgg**ggatcc**CTGGCCAGAATATGTGCGTCG | |
| pROBE-*vfmF*-F | ggaattggggatcgg**aagctt**CGAATTCAGTGGTTGATTATTGTCC | |
| pROBE-*vfmF*-R | gagctcggtacccgg**ggatcc**GGCATGCGTTTCAATTTCATC | |
| pROBE-*vfmK*-F | ggaattggggatcgg**aagctt**CGCCACCATGATGCCATC | |
| pROBE-*vfmK*-R | gagctcggtacccgg**ggatcc**CAGCGCGTCAGGCTGGGT | |
| pROBE-*ohrR*-F | ggaattggggatcggaagcttCGAACCCGAACTCCGCTT | |
| pROBE-*ohrR*-R | gagctcggtacccggggatccCAGAAACCGTTACCCCATCG | |
| pET30a -F | TAATACGACTCACTATAGGG | |
| pET30a -R | GCTAGTTATTGCTCAGCGG | |
| pET30a-*emrR*-BamHI-F | gccatggctgatatc**ggatcc**ATGGAAAGTTCGTTCGCTCCA | |
| pET30a-*emrR*-HindIII-R | ctcgagtgcggccgc**aagctt**TTAGAAACCGTCCTGCTCCATG | |
| pCold-*ohrR*-F | ctcggtaccctcgag**ggatcc**GAGCGATATAGAGTGGCAGCCA | |
| pCold-*ohrR*-R | tgcaggtcgac**aagctt**TTACATCATATCGACTCATCAAATATGTC | |
| pCold-SUMO-F | CCCCTGAAGATTTGGACATG | |
| pCold-SUMO-F | TGGCAGGGATCTTAGATTCTG | |
| *∆vfmE*(*uidA*)-F | CGACATTTTCCGCGATGGTC | |
| *∆vfmE*(*uidA*)-R | AGCGGTGGTTTGGTGTGTAG | |
| *∆vfmE*(*uidA*)-1 | cccctgcaggtcgac**ggatcc**TCACGGTTTCGCAGTCGAG | |
| *∆vfmE*(*uidA*)-2 | gggtttctacaggacgtaaGTTAAAAATATCGTTATCTACGGTATGGG | |
| *∆vfmE*(*uidA*)-3 | cTTACGTCCTGTAGAAACCCCAAC | |
| *∆vfmE*(*uidA*)-4 | gttaTTGTTTGCCTCCCTGCTGC | |
| *∆vfmE*(*uidA*)-5 | gcagggaggcaaacaaTAACTCAAAGCCCGCCATGA | |
| *∆vfmE*(*uidA*)-6 | cggactatagactat**actagt**GCTGCTGGCCAGTCCGGA | |
| *β*-Galactosidase activity detection | | |
| pLME-*emrR*-F | ttaggcaccccaggc**ggatcc**GAGACACCATGAATACATCAGTATTATTG | |
| pLME-*emrR*-R | ggaaacagtggacat**ggatcc**GTCTTCGATTGGAGCGAACG | |
| pLME-*ohrR*-F | ttaggcaccccaggcg**gatcc**CGAACCCGAACTCCGCTT | |
| pLME-*ohrR*-R | ggaaacagtggacat**ggatcc**ATCACTCAGCATCGGTTTGGTAT | |
| RT-qPCR |  | |
| 16s1369F | CGGTGAATACGTTCYCGG | |
| 16s1541R | AAGGAGGTGATCCRGCCGCA | |
| q*vfmA*-F | GAGTTATGCCGTTTCATT | |
| q*vfmA*-R | GTCCGAACAATACAGATG | |
| q*vfmE*-F | GACAATATCGGCAACAATA | |
| q*vfmE*-R | GTTGAAGAGACGACAGATA | |
| q*vfmF*-F | TGGTTCAACTTGATAATGT | |
| q*vfmF*-R | CGAGTGAATAGAAAGGTTT | |
| q*vfmK*-F | CGGCTATGTAGAAGAGAT | |
| q*vfmK*-R | CGCCTTCAGAGAATAGTA | |
| q*emrR*-F | TTGCTGCTGCGTTTGTTC | |
| q*emrR*-R | TCGTTGATGTCCTGCTCTT | |
| EMSA probe |  | |
| EmrR-F | TGCAGTACCCGGAAAGACAG | |
| EmrR-R | GGAGCGAACGAACTTTCCATAGA | |
| VfmA-F | TTCGTTCATTGAGGACCACG | |
| VfmA-R | CACGGCTATCGTCCCCTTATC | |
| VfmE-F | TTTCCATCCCGCTTTATCCC | |
| VfmE-R | TCATGGTGTCATCCCAAAAAAATAG | |
| VfmF-F | GTGTGCCATGTTGTTATTGC | |
| VfmF-R | CATGGGAAATCCTCAGTGTATAAATAG | |
| VfmK-F | AAATCACCTGTAAACGCAGC | |
| VfmK-R | CATGATGAAGCCGCTCACG | |
| * The underlines are the site of restriction endonuclease | | |
