# Supplementary material for: A Hierarchical OhrR–EmrR Regulatory Circuit Fine‐Tunes the Vfm Quorum‐ Sensing System and Virulence in Dickeya oryzae
Source: Mol Plant Pathol. 2026 Oct 19;27(10):e70354. doi: 10.1111/mpp.70354 (PMC13680859; doi:10.1111/mpp.70354)
Supplement: Supplementary file 2 [file MPP-27-e70354-s002.docx]

Fig. S1 Specific competitive EMSA assays for OhrR and EmrR binding to the *vfmK* promoter, with the *pelE* promoter fragment serving as a negative control.
