# Supplementary material for: A Hierarchical OhrR–EmrR Regulatory Circuit Fine‐Tunes the Vfm Quorum‐ Sensing System and Virulence in Dickeya oryzae
Source: Mol Plant Pathol. 2026 Oct 19;27(10):e70354. doi: 10.1111/mpp.70354 (PMC13680859; doi:10.1111/mpp.70354)
Supplement: Supplementary file 5 [file MPP-27-e70354-s005.docx]

Fig. S2 Localization of OhrR and EmrR binding motifs within the *vfmA*, *vfmE*, *vfmF* and *vfmK* promoter regions. Schematic representation showing the positions of identified OhrR and EmrR binding motifs upstream of the translation star sites of *vfmA*, *vfmE*, *vfmF* and *vfmK*.
