# Supplementary material for: A Hierarchical OhrR–EmrR Regulatory Circuit Fine‐Tunes the Vfm Quorum‐ Sensing System and Virulence in Dickeya oryzae
Source: Mol Plant Pathol. 2026 Oct 19;27(10):e70354. doi: 10.1111/mpp.70354 (PMC13680859; doi:10.1111/mpp.70354)
Supplement: Supplementary file 6 [file MPP-27-e70354-s003.docx]

Fig. S5 EMSA assays using the *vfmA* promoter probe with OhrR and EmrR at different molar ratios.
